# A comparative phytochemical study of nine Lauraceae species by using chemometric data analysis

**DOI:** 10.1371/journal.pone.0273616

**Published:** 2022-09-09

**Authors:** Mira Oh, Hyun-Seung Park, Soohyun Um, Tae-Jin Yang, Seung Hyun Kim

**Affiliations:** 1 College of Pharmacy, Yonsei Institute of Pharmaceutical Sciences, Yonsei University, Incheon, Korea; 2 Department of Agriculture, Forestry and Bioresources, Research Institute of Agriculture and Life Sciences, and Plant Genomics and Breeding Institute, College of Agriculture and Life Sciences, Seoul National University, Seoul, Republic of Korea; Universidade Católica Portuguesa Escola Superior de Biotecnologia: Universidade Catolica Portuguesa Escola Superior de Biotecnologia, PORTUGAL

## Abstract

The diversity of secondary metabolites of individual plants results from multiple enzymatic processes *in planta* and various environmental factors, such as temperature, moisture, and soil conditions. Chemical composition analysis of plants can lead to a new method to understand relationship among comparable plants along with biological classification such as genetic and anatomical method. In this study, the chemical diversity of nine different Lauraceae species was investigated, and the plant samples were chemically analyzed and classified. Multivariate analysis methods, such as PLS-DA, were used to select important metabolites distinguishing the nine Lauraceae species. The selected metabolites were identified through preparative LC-MS or MS/MS fragment pattern analysis. In addition, the chemical dendrogram for the nine Lauraceae species was interpreted through molecular network analysis and compared with the genetic dendrogram. This approach enabled us to compare the complete chemical compositions of multiple plant samples to identify relationships among plants.

## Introduction

Individual plants are chemically rich with diverse chemical compositions regardless of their genus or phenotype [1]. The chemical diversity of plant metabolites has been reported to be one of the phenotypic results of evolution [2]. We can gain insights into the evolution and taxonomy of different plant species from the distribution of specialized metabolites [3]. Although phytochemical studies are recognized for being able to deepen the understanding of relationships among targeted plants, comparative chemical analyses among Lauraceae have not been performed [4, 5]. Many taxonomic analyses for Lauraceae species have been conducted on using morphological [6, 7], anatomical [8–11], and genetic [12–14] differences to distinguish species.

The Lauraceae family is composed of approximately 55 genera totalling over 3000 species distributed throughout Southeast Asia and tropical America [15]. Phytochemicals of the Lauraceae family are used in various industrial fields and worthy of investigation. This family contains valuable secondary metabolites and is therefore has economically important the food, pharmaceutical, and perfumery industries [16, 17]. For example, *Cinnamomum burmannii*, *Litsea pungens*, and *Laurus nobilis* are used in cooking as spices, and the fruits of *Persea americana* Mill. are used as a food ingredient [5, 15]. The essential oil, a representative component of the Lauraceae family, is both used as a perfume ingredient and has a wide range of biological activities, such as cytotoxic, antimicrobial, antioxidant, and anti-inflammatory activities [18–22].

Metabolomics is a useful tool for comparing complete metabolites and differentiating genotypes of individual plants in a rapid and unbiased way [23]. The scope of metabolomics has been considerably expanded as an important method of fingerprinting and profiling for primary and secondary metabolites, as well as for the selection and identification of targeted metabolites [24]. Liquid chromatography‒mass spectrometry (LC-MS) is a widely used technique in plant metabolomics. A high sensitivity and strong compatibility with biomolecules make LC-MS suitable for profiling chemical composition from a large quantity of data [25, 26]. Metabolomics with multivariate analysis techniques is useful for nontargeted metabolic profiling [27]. For large-scale datasets, multivariate analyses, such as principal component analysis (PCA) and partial least squares discriminant analysis (PLS-DA), can be used to simplify complex data by transforming high-dimensional raw mass data to a lower number of variables as points in maps [28]. This statistical method enables the chemical compositions of plant metabolites to be conveniently discriminated. Specifically, the VIP score of PLS-DA can be determined by measuring the importance of individual metabolites in samples to improve the classification accuracy of samples [29].

Fourteen species in seven genera of the Lauraceae family are found in Korea that are also native to Japan and Taiwan. In this study, nine Korean endemic Lauraceae species were compared and classified by their metabolite composition. The LC-MS spectral data of the nine samples were applied to multivariate analyses. Then, the variable importance in projection (VIP) scores determined using PLS-DA were used to select specific metabolites that are key factors for discriminating samples. The selected discriminatory metabolites were identified by using NMR or comparison with MS/MS fragment patterns obtained from spectral databases, such as GNPS (Global Natural Products Social Molecular Networking). The complete chemical composition of the samples was visualized in a molecular networking system and compared with a chemical dendrogram. We used this approach to classify nine Lauraceae species based on their chemical composition, and the chemical dendrogram was compared with phylogenomic results obtained from a plastid genome sequence analysis.

## Materials and methods

### Sample preparation

Leaves of nine Lauraceae samples were collected from Jeju, Korea (between August 2^nd^ and 8^th^, 2020), and authenticate by Dae Yang Park of Korea Medicinal Plant Farming Corporation. A voucher specimen (YU2020CC/CY/LDE/LC/LJ/MJ/MY/NA/NS) was deposited in the Herbarium at the College of Pharmacy, Yonsei Institute of Pharmaceutical Sciences, Yonsei University, Incheon, Korea. The samples were dried at 40°C for 48 h and homogenized. A powder of each leaf type (50 mg) was transferred to a 5 mL glass vial, to which 1 mL of 50% MeOH was added. The samples were then sonicated for an hour at room temperature. The supernatant was filtered using a 0.2 μm pore syringe filter (Whatman, Clifton, USA). The filtrate was completely evaporated under a nitrogen flow and stored at −20°C until analysis. The species information and codes of the nine Lauraceae samples used in this study are shown in Table 1.

**Table 1:** Information for the nine Lauraceae species used in this experimental study.

Family	Genus	Species	Location	Code
Lauraceae	*Cinnamomum*	*C*. *camphora* (L.) J.Presl	Jeju, Korea	CC
		*C*. *yabunikkei* H.Ohba		CY
	*Lindera*	*L*. *erythrocarpa* Makino	Jeju, Korea	LDE
	*Litsea*	*L*. *coreana* H.Lév.	Jeju, Korea	LC
		*L*. *japonica* (Thunb.) Jussieu		LJ
	*Machilus*	*M*. *japonica* Siebold & Zucc.	Jeju, Korea	MJ
		*M*. *thunbergii* Siebold & Zucc.		MT
	*Neolitsea*	*N*. *aciculata* (Blume) Koidz.	Jeju, Korea	NA
		*N*. *sericea* (Blume) Koidz.		NS

### UPLC-QTOF-MS analysis

The concentrated samples were dissolved in 50% MeOH (JT Baker, Phillipsburg, USA) to a concentration of 1 mg/mL in preparation for LC-MS analysis. The analysis was performed on a UPLC-QTOF-MS analytical system consisting of an Agilent 1290 Infinity LC (Agilent Technologies, Palo Alto, USA) and an Agilent 6550 iFunnel QTOF LC-MS equipped with a dual Agilent Jet Stream (AJS) ESI source. The UPLC column was a YMC-Triart C18 column (2.0 × 150 mm, 1.9 μm; YMC KOREA Co., Seongnam, Korea) that was maintained at 25°C during the analysis. The mobile phases were 0.1% formic acid in water (A) and 0.1% formic acid in acetonitrile (B), and the following gradients were used: 10–30% B (0–15 min), 30–50% B (15–17 min), 50–80% B (17–20 min), 80–100% B (20–20.1 min), 100% B (20.1–25 min), 100–10% B (25–25.1 min), and 10% B (25.1–28 min). The flow rate was 0.4 mL/min. Each sample was injected in six 10 μL replicates, and a blank (50% MeOH) was injected at the beginning of the sample sequence.

The MS experiment was performed with a dual AJS ESI source under the following conditions: drying gas temperature 300°C, drying gas flow 8 L/min, nebulizer gas pressure 35 psi, sheath gas temperature 350°C, sheath gas flow 11 L/min, and capillary voltage +3.5 kV for the positive ionization mode. The QTOF parameters were set to an acquisition rate of five spectra/sec for MS (mass range of 100−3200 *m/z*) and three spectra/sec for MS/MS (mass range of 100−3200 *m/z*). The collision energy for fragmentation was set to 20, 40, and 60 eV. To obtain the exact mass, calibration was performed with an Agilent tune mix (Agilent Technologies, Palo Alto, USA) from 100 to 1600 Da. Data were acquired in centroid mode at high resolution (4 GHz).

### Data preprocessing

For chemometric analysis, the mass features were detected from the LC-MS raw data using MZmine 2.53 under the following conditions: a retention time range of 0–20 min; a mass detection noise level of 1500 for MS1 and 20 for MS2; a minimum time span of 0.01 min, a minimum height of 5000 and an *m/z* tolerance of 0.001 *m/z* for the chromatogram builder; a baseline cut‐off algorithm with a minimum peak height of 10,000, a peak duration range of 0.01–0.5 min, and a baseline level of 500 for chromatogram deconvolution; an isotopic peak-grouper algorithm with an *m/z* tolerance of 0.006 and a retention time tolerance of 0.15 min; a join aligner module with an *m/z* tolerance of 0.01, an absolute retention time tolerance of 0.3 min, an *m/z* weight of 70, and a retention time weight of 30. Duplicate peaks with blanks were manually removed from the aligned peak table.

### Chemometric data analysis

Multivariate analysis was performed using MetaboAnalyst 5.0 (http://www.metaboanalyst.ca) software [30]. The preprocessed peak intensity table was uploaded, and then PCA and PLS-DA were performed using the R package’s prcomp and plsr functions, respectively. A list of important metabolites distinguishing nine Lauraceae species was generated by PLS-DA using the VIP score as a measure. A hierarchical clustering dendrogram was obtained using the mass feature matrix, functioned by hclust of the stats R package. The measure of the Euclidean distance was used in conjunction with Ward’s clustering algorithm to generate the dendrogram.

To perform molecular networking, the raw mass spectral data were converted into mzML file formats using MSConvert 3.0 and uploaded to the GNPS server. The tolerances for the precursor and product ions were set to 2.0 and 0.5 Da, respectively. A network was generated using the MS-Cluster algorithm, with parameter settings of minimum cosine pairs of 0.7, minimum matched fragment ions of 6, and a minimum cluster size of 2. The molecular networking job on GNPS can be found at https://gnps.ucsd.edu/ProteoSAFe/status.jsp?task=1d5650eba32b484aa176f75adc7c02d4.

### Metabolite identification

Preparative LC-MS (prep LC-MS) was carried out using an Agilent 1100 series capillary LC system (Agilent Technologies, Palo Alto, USA) coupled with a Waters micromass ZQ mass spectrometer (Waters Co., Milford, USA). The prep column was a YMC-Triart C18 semiprep column (10.0 × 150 mm, 5 μm; YMC KOREA Co., Seongnam, Korea). The mobile phases were 0.1% formic acid in water and 0.1% formic acid in acetonitrile with the same solvent gradients used in the QTOF-LC-MS analysis. Broad fractions containing each target peak were eluted from the samples (50% MeOH extract), followed by separating the single peaks more carefully under isocratic solvent conditions. Compound **2** was obtained from the *Neolitsea sericea* (Blume) Koidz. extract, **4** and **9** were obtained from *Litsea coreana* H. Lév., **6** was obtained from *Machilus japonica* Siebold & Zucc., and **13** was obtained from *Lindera erythrocarpa* Makino (the compound names corresponding to the compound numbers are presented in Table 2). All the NMR spectra of the isolated compounds obtained using prep LC-MS were recorded on a JEOL JNM-ECZ600R spectrometer (JEOL, Tokyo, Japan) operated at 600 and 150 MHz for hydrogen and carbon, respectively. The chemical shifts are reported in parts per million from tetramethylsilane. Data processing was carried out by the MestReNova ver.12.0.1 program.

**Table 2:** List of fourteen metabolites selected as important features for sample discrimination.

No.	Compound name	VIP score	Class	Formula	RT^a^ (min)	m/z	Adduct ion	MS/MS fragment
1	neochlorogenic acid	4.8429	cinnamic acids derivatives	C_16_H_18_O_9_	3.42	355.0999	M+H	215, 185, 163
2	afzelin^b^	4.3162	flavonoids	C_21_H_20_O_10_	15.25	433.1118	M+H	-
3	laurolitsine	4.028	isoquinoline alkaloids	C_18_H_19_NO_4_	6.83	314.1372	M+H	297, 265, 237
4	catechin^b^	3.9379	flavonoids	C_15_H_14_O_6_	5.72	291.0927	M+H	147, 139, 123
5	unidentified 1	3.7352	-	-	7.18	563.1522	-	378, 313, 223, 123
6	chlorogenic acid^b^	3.2855	cinnamic acids derivatives	C_16_H_18_O_9_	5.40	355.1026	M+H	215, 185, 163
7	coclaurine	3.1933	isoquinoline alkaloids	C_17_H_19_NO_3_	6.66	286.1462	M+H	269, 237, 175, 107
8	dihydrokaempferol	3.0558	flavonoids	C_15_H_12_O_6_	11.78	289.0710	M+H	215, 153, 149, 107
9	epicatechin^b^	2.7646	flavonoids	C_15_H_14_O_6_	7.35	291.0845	M+H	139, 123
10	unidentified 2	2.7263	-	-	2.82	188.0695	-	-
11	roemerine	2.7097	isoquinoline alkaloids	C_18_H_17_NO_2_	15.95	280.1321	M+H	249, 219
12	phenylalanine	2.695	amino acids	C_9_H_11_NO_2_	2.22	166.0854	M+H	120, 103
13	quercitrin^b^	2.5482	flavonoids	C_21_H_20_O_11_	13.08	449.1064	M+H	-
14	unidentified 3	2.5469	-	-	19.77	281.2104	-	-

^a^ Retention time

^b^ Identified using NMR (the NMR data are shown in S3–S12 Figs in S1 File)

Compounds **1**, **3**, **7**, **8**, **11**, and **12** were putatively annotated by comparison with masses, chemical formulas, and MS/MS fragment spectra from chemical databases (Table 2). The exact mass and molecular formulas were calculated by MassHunter qualitative analysis software B.06.00 (Agilent Technologies, Palo Alto, USA) with a mass accuracy below 7.5 ppm. The accurate mass and chemical formulas were used to search the compounds in each sample in the Scifinder® chemical database (http://www.scifinder.org) (Compounds **1**, **8**, and **12** were identified in the *L*. *erythrocarpa* Makino database, **3** was identified in the *L*. *japonica* (Thunb.) Jussieu database, and **7** and **11** were identified in the *N*. *sericea* (Blume) database). Finally, our MS/MS fragment data for the target compounds were compared to the GNPS spectral database or other literature.

### DNA extraction and barcoding analysis

Each leaf sample was ground with liquid nitrogen using a mortar and pestle. DNA was extracted using a GeneAll Plant SV mini kit (GeneAll, Seoul, Korea) following the manufacturer’s instructions. DNA barcoding analysis was performed using universal primers to amplify each *trnH-GUG* and *rbcL* region under conditions suggested in a previous paper [31]. Amplicons were sequenced using ABI 3730xL (NICEM, Seoul, Korea). After combining the sequences from two regions, pairwise alignment was performed by multiple alignment using the fast Fourier transform, with the plastid genomes of each species collected from GenBank for making consensus sequences. A neighbor-joining phylogenetic tree was drawn by MEGA X with 1000 bootstrap replicates.

## Results and discussion

### LC-MS profiles

LC-MS total ion current (TIC) chromatograms were acquired from the leaves of nine Lauraceae samples under the optimized UPLC-QTOF-MS conditions (S1 Fig in S1 File). The peaks were detected more sensitively in positive ionization mode than in negative mode. Additionally, the chromatograms showed a higher peak abundance when 0.1% formic acid was added to the mobile phase. S1 Fig in S1 File shows very different MS patterns for the nine Lauraceae species even though the species belong to the same genus. The species information and codes of the nine Lauraceae samples used in this study are shown in Table 1.

### Chemometric analyses

**Fig 1:**
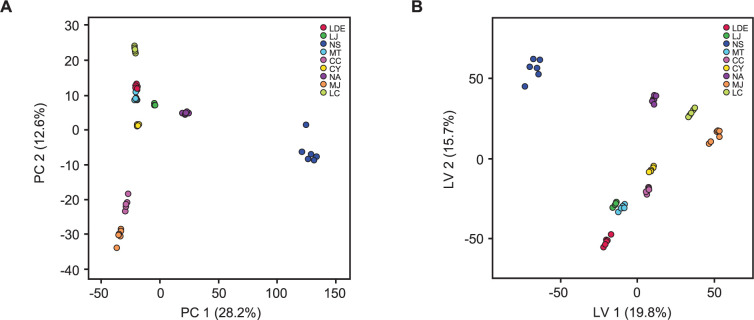
Multivariate statistical analysis plots of nine Lauraceae species based on LC-MS spectral data. (**A**) PCA score plot. (**B**) PLS-DA score plot. LDE, *Lindera erythrocarpa* Makino; LJ, *Litsea japonica* (Thunb.) Jussieu; NS, *Neolitsea sericea* (Blume) Koidz.; MT, *Machilus thunbergii* Siebold & Zucc.; CC, *Cinnamomum camphora* (L.) J. Presl; CY, *C*. *yabunikkei* H. Ohba; NA, *N*. *aciculata* (Blume) Koidz.; MJ, *M*. *japonica* Siebold & Zucc.; and LC, *L*. *coreana* H.Lév.

**Fig 2:**
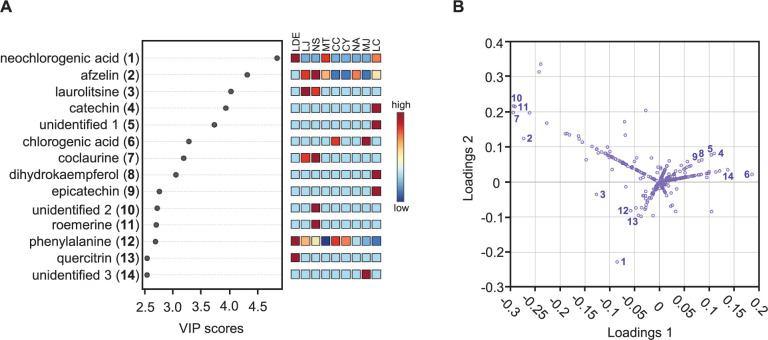
VIP scores and loading plot obtained using the PLS-DA model for nine Lauraceae metabolites. (**A**) Important metabolites identified by PLS-DA. The colored boxes on the right indicate the relative concentrations of the corresponding metabolite in each group under study. (**B**) Loading plot showing PC1-PC2. The 14 important metabolites selected according to the VIP scores are indicated on the corresponding plots. LDE, *Lindera erythrocarpa* Makino; LJ, *Litsea japonica* (Thunb.) Jussieu; NS, *Neolitsea sericea* (Blume) Koidz.; MT, *Machilus thunbergii* Siebold & Zucc.; CC, *Cinnamomum camphora* (L.) J. Presl; CY, *C*. *yabunikkei* H. Ohba; NA, *N*. *aciculata* (Blume) Koidz.; MJ, *M*. *japonica* Siebold & Zucc.; and LC, *L*. *coreana* H.Lév.

**Fig 3:**
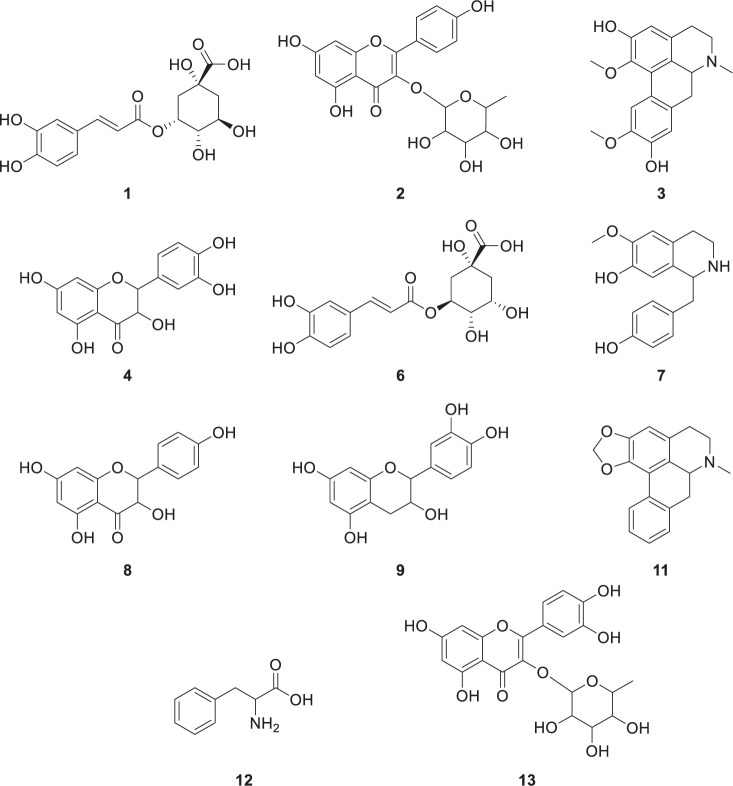
Representative chemical structures of eleven metabolites contributing to sample discrimination. (**1**) neochlorogenic acid; (**2**) afzelin; (**3**) laurolitsine; (**4**) catechin; (**6**) chlorogenic acid; (**7**) coclaurine; (**8**) dihydrokaempferol; (**9**) epicatechin; (**11**) roemerine; (**12**) phenylalanine; and (**13**) quercitrin.

**Fig 4:**
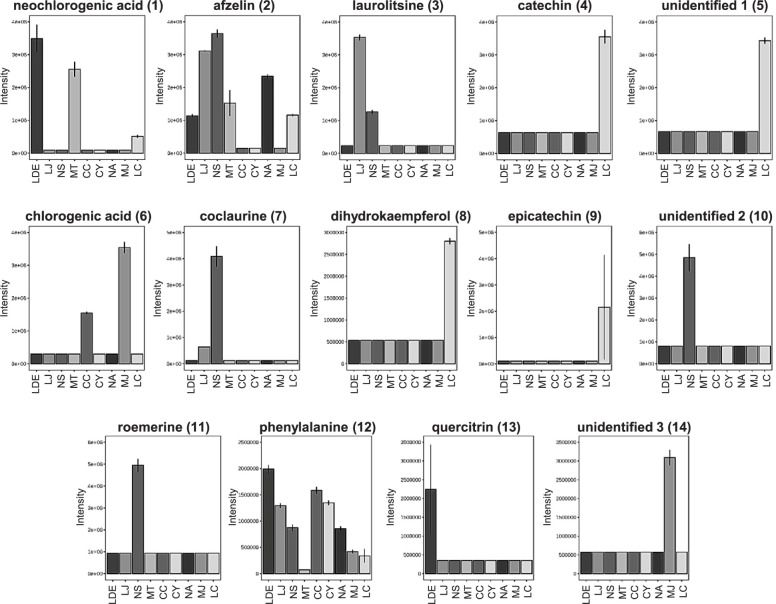
Bar plots of fourteen metabolites selected as important features according to the VIP scores. The bar plots show the intensity of the corresponding ions in the nine analyzed Lauraceae samples. LDE, *Lindera erythrocarpa* Makino; LJ, *Litsea japonica* (Thunb.) Jussieu; NS, *Neolitsea sericea* (Blume) Koidz.; MT, *Machilus thunbergii* Siebold & Zucc.; CC, *Cinnamomum camphora* (L.) J. Presl; CY, *C*. *yabunikkei* H. Ohba; NA, *N*. *aciculata* (Blume) Koidz.; MJ, *M*. *japonica* Siebold & Zucc.; and LC, *L*. *coreana* H.Lév.

A mass peak list was produced from the LC-MS spectral data of each sample by using MZmine prefiltering. A total of 447 mass features were uploaded to a MetaboAnalyst server to perform multivariate statistical analyses on the chemical differences among the tested Lauraceae species. Score plots generated from both the PCA and PLS-DA models simply visualized information-rich spectral data by reducing the dimensions. PLS-DA is a supervised method that is an alternative to the unsupervised PCA method and interprets data with intergroup variability to better represent group structures [27]. The nine Lauraceae species were clustered more clearly in the PLS-DA (Fig 1B) than in the PCA (Fig 1A). The cross-validated coefficients of the PLS-DA model were Q^2^ = 0.985 and R^2^ = 0.99593, indicating good model quality (S2 Fig in S1 File). The compounds showing a significant difference among the nine Lauraceae samples were selected using the PLS-DA model (VIP score > 2.5), and a PLS-DA loading plot was generated to obtain the distribution of the selected compounds for all the compounds in the samples. The loading scatter plot of the PLS-DA model indicates the relationship between a characteristic variable and a categorical variable, which reflects the contribution of a considered variable on the score plot. The fourteen selected metabolites (Fig 2A) (representative chemical structures of the selected metabolites are shown in Fig 3) are displayed on the loading plot (Fig 2B), indicating that chemical compounds, such as cinnamic acid derivatives, flavonoids, and isoquinoline alkaloids, were the major features distinguishing the nine Lauraceae species. Isoquinoline alkaloids, such as coclaurine (**7**) and roemerine (**11**), were selected as marker metabolites to separate *Neolitsea sericea* (Blume) Koidz (NS) from the other species on the PLS-DA score plot because of the relatively high concentrations of **7** and **11** in NS. The bar plot data presented in Fig 4 show that **7** was also found in small quantities in LJ, whereas **11** was only detected in NS and could therefore be a more definitive marker than **7**. Flavonoids, such as catechin (**4**), dihydrokaempferol (**8**), and epicatechin (**9**), can similarly be used as differential markers to distinguish *Litsea coreana* H. Lév. (LC). *Lindera erythrocarpa* Makino (LDE) contained an abundance of neochlorogenic acid (**1**), phenylalanine (**12**), and quercitrin (**13**) and *Machilus japonica* Siebold & Zucc. (MJ) contained an abundance of chlorogenic acid (**6**). The fourteen metabolites are ranked by their VIP scores in Table 2, and the relative peak height of the metabolites are represented by using box and whisker plots in Fig 4.

**Fig 5:**
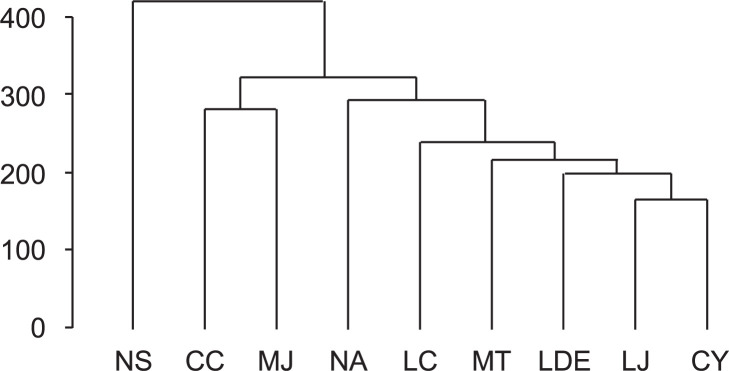
Chemical dendrogram of nine Lauraceae species. A dendrogram was generated based on the chemical components of the samples using the Euclidean distance and Ward’s clustering algorithm. LDE, *Lindera erythrocarpa* Makino; LJ, *Litsea japonica* (Thunb.) Jussieu; NS, *Neolitsea sericea* (Blume) Koidz.; MT, *Machilus thunbergii* Siebold & Zucc.; CC, *Cinnamomum camphora* (L.) J. Presl; CY, *C*. *yabunikkei* H. Ohba; NA, *N*. *aciculata* (Blume) Koidz.; MJ, *M*. *japonica* Siebold & Zucc.; and LC, *L*. *coreana* H.Lév.

Hierarchical clustering analysis (HCA) was performed to investigate the grouping patterns of nine Lauraceae species according to the corresponding phytochemicals (Fig 5). The distinction between samples was confirmed by unsupervised HCA of the mass feature matrix. The dendrogram consisted of one separated branch (NS) and two clusters, of which one comprised CC and MJ and the other comprised NA, LC, MT, LDE, LJ, and CY. Although CC was clustered with MJ in the same cluster at a short phytochemical distance, CC and CY showed relatively weak correlations despite belonging to the same genus. Similarly, NA was more closely related to different genera, such as LC, MT, LDE, LJ, and CY, than to the same genera, such as NS. In the PCA score plots, NS was strongly separated from the other samples along the PC1 axis (28.2%), and CC and MJ were also separated along the PC2 axis (12.6%), corresponding with the HCA result (Fig 1A). Considering these results together with the HCA results indicates that the close chemical compositions of the nine Lauraceae species were not identical with their genomic closeness. He et al. (2014) [32] similarly observed inconsistencies between the chemical taxonomy and molecular phylogeny of four *Coptis* species. Wen et al. (2020) [33] also found that the chemical classification of *Nardostachys jatamansi* collected from different habitats was inconsistent with molecular phylogenetic analysis results. Environmental factors and developmental conditions can affect phytochemical synthesis *in planta* and the accumulation of metabolic constituents [34]. In nature, plant secondary metabolism pathways elicit an array of plant defensive compounds called secondary metabolites. A secondary metabolite is synthesized in organs or tissues in response to various environmental stimuli, such as light, temperature, soil conditions, and microbiota [35]. Accordingly, the corresponding genes to each plant secondary metabolite are regulated at the transcriptional level by multiple transcription factors and not only by the genetic structure [36]. Therefore, the difference between our chemical and gene taxonomic results can be explained by environmental factors affecting the biosynthesis of metabolic compounds in plants.

**Fig 6:**
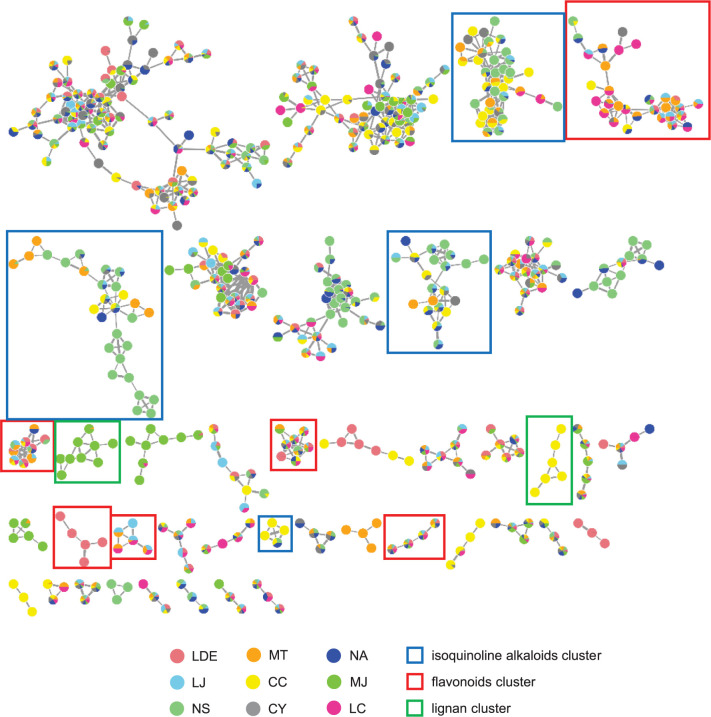
The networking analysis results of the nine Lauraceae species. A network was generated using MS/MS spectra through classical molecular networking on the GNPS server and visualized with nodes and edges through Cytoscape 3.8.0. The nodes consist of pie charts based on the peak intensity proportion for each metabolite. The thickness of the edges was determined by the similarity between two connected nodes with edge widths ranging from 6.0 to 16.0. The blue, red, and green boxes indicate isoquinoline alkaloids, flavonoids, and lignin clusters, respectively. LDE, *Lindera erythrocarpa* Makino; LJ, *Litsea japonica* (Thunb.) Jussieu; NS, *Neolitsea sericea* (Blume) Koidz.; MT, *Machilus thunbergii* Siebold & Zucc.; CC, *Cinnamomum camphora* (L.) J. Presl; CY, *C*. *yabunikkei* H. Ohba; NA, *N*. *aciculata* (Blume) Koidz.; MJ, *M*. *japonica* Siebold & Zucc.; and LC, *L*. *coreana* H.Lév.

**Fig 7:**
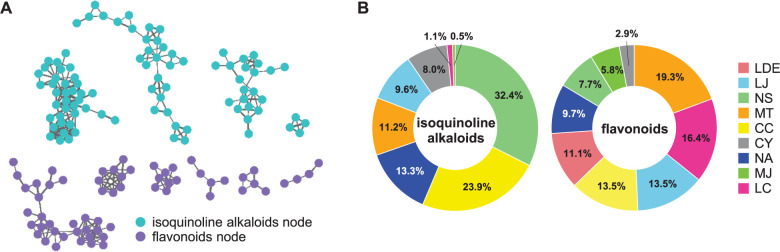
The chemical composition of Lauraceae samples based on the network analysis. (A) Clusters annotated as isoquinoline alkaloids or flavonoids through classical molecular networking. The thickness of the edges was determined by the similarity between two connected nodes with edge widths ranging from 6.0 to 16.0. (B) The node composition ratio of isoquinoline alkaloids or flavonoids in each sample. LDE, *Lindera erythrocarpa* Makino; LJ, *Litsea japonica* (Thunb.) Jussieu; NS, *Neolitsea sericea* (Blume) Koidz.; MT, *Machilus thunbergii* Siebold & Zucc.; CC, *Cinnamomum camphora* (L.) J. Presl; CY, *C*. *yabunikkei* H. Ohba; NA, *N*. *aciculata* (Blume) Koidz.; MJ, *M*. *japonica* Siebold & Zucc.; and LC, *L*. *coreana* H.Lév.

A molecular network was constructed to investigate the complete chemical composition of the nine Lauraceae species. A molecular network is a spectral analysis tool for grouping various compounds by their fragmentation patterns. In GNPS, each MS/MS spectrum is aligned in a dataset, and structurally related molecules are clustered by using the MS-Cluster algorithm [37]. A molecular network of nine Lauraceae species was generated using GNPS and visualized through Cytoscape 3.8.0, an open-source software for visualizing complex networks (Fig 6). A total of 1414 nodes and 73 clusters from the nine samples were detected by the GNPS analysis (Fig 6): four isoquinoline alkaloid clusters (91 nodes in total), six flavonoid clusters (61 nodes in total), and two lignan clusters (15 nodes in total). The annotated isoquinoline alkaloid and flavonoid clusters (shown as blue and red squares in Fig 6) were composed of the nodes from eight (LJ, NS, MT, CC, CY, NA, MJ, and LC) and nine (LDE, LJ, NS, MT, CC, CY, NA, MJ, and LC) samples, respectively. However, the nodes in the lignan clusters were only detected from MJ, MT, and CC. For the detected isoquinoline alkaloid clusters, the node composition ratio was the highest in NS (32.4%), followed by CC (23.9%) and NA (13.3%), whereas MJ accounted for the lowest ratio at 0.5%. Under our analysis conditions, no isoquinoline alkaloid nodes were detected in the LDE sample (Fig 7A and 7B). Flavonoid nodes were detected for all nine Lauraceae species. MT had the highest ratio of flavonoid nodes at 19.3%, followed by LC (16.4%) and LJ (13.5%). CY accounted for the lowest ratio at 2.9% (Fig 7A and 7B). According to the molecular network results, NS, which was the only sample separated from the others in the chemical dendrogram (Fig 5), has the highest isoquinoline alkaloid contents among the samples (Fig 6). In addition, CC and MJ, which were grouped into one cluster in the chemical dendrogram (Fig 5), contained most of the lignan compounds detected in the network analysis (Fig 6).

### Metabolite identification

In this study, metabolites were identified by using extensive spectroscopic methods or verified through a literature search based on various spectral databases. In Table 2, the five compounds annotated with ‘b’ (Compounds **2**, **4**, **6**, **9**, and **13**) were isolated by using prep LC-MS, and the chemical structures of the purified compounds were confirmed through NMR. Prep LC-MS is an effective and highly efficient approach to purify small molecules [38]. Unlike UV-based separation with HPLC, this technique enables selective isolation of specific compounds with exact masses, thereby eliminating the need for additional purification analysis to determine the mass of the isolated compounds [39]. The purified compounds were compared with reported ^1^H and ^13^C NMR and MS data and identified as afzelin (**2**) [40], catechin (**4**) [41], chlorogenic acid (**6**) [42], epicatechin (**9**) [41], and quercitrin (**13**) [42] (the NMR data are shown in S3–S12 Figs in S1 File).

Compounds **1**, **3**, **7**, **8**, **11**, and **12** were putatively identified as neochlorogenic acid (**1**), laurolitsine (**3**), coclaurine (**7**), dihydrokaempferol (**8**), roemerine (**11**), and phenylalanine (**12**), respectively, based on a literature search and comparison of MS/MS patterns [43–46]. The exact mass, chemical formula, retention time, and MS/MS fragment information of the compounds were obtained from the UPLC-QTOF-MS data using MassHunter software. Neochlorogenic acid (**1**) was expected to be structurally similar to chlorogenic acid (**6**) because the mass and MS/MS spectrum were the same as those of **6**, except for the retention time (Table 2). According to the data from Kurita et al. (2016) [47], the retention times of neochlorogenic acid (8.11 min) and chlorogenic acid (16.67 min) were clearly different under the respective HPLC analysis conditions, despite having the same mass and chemical formula. According to our analysis, the two compounds were detected at different retention times: Compound **1** (3.42 min) was detected earlier than Compound **6** (5.40 min) (S13 Fig in S1 File). Considering the results together, Compound **1** was putatively identified as neochlorogenic acid (**1**).

Compounds **5**, **10**, and **14** were purified by using prep LC-MS at the beginning; however, their yields were too low to obtain NMR data. In addition, MS/MS pattern comparison was not possible because the MS/MS fragment information of the compounds could not be obtained through QTOF-LC-MS analysis and there was no match in a chemical database with **5**, **10**, and **14**.

### DNA barcoding and genetic relationship of Lauraceae

**Fig 8:**
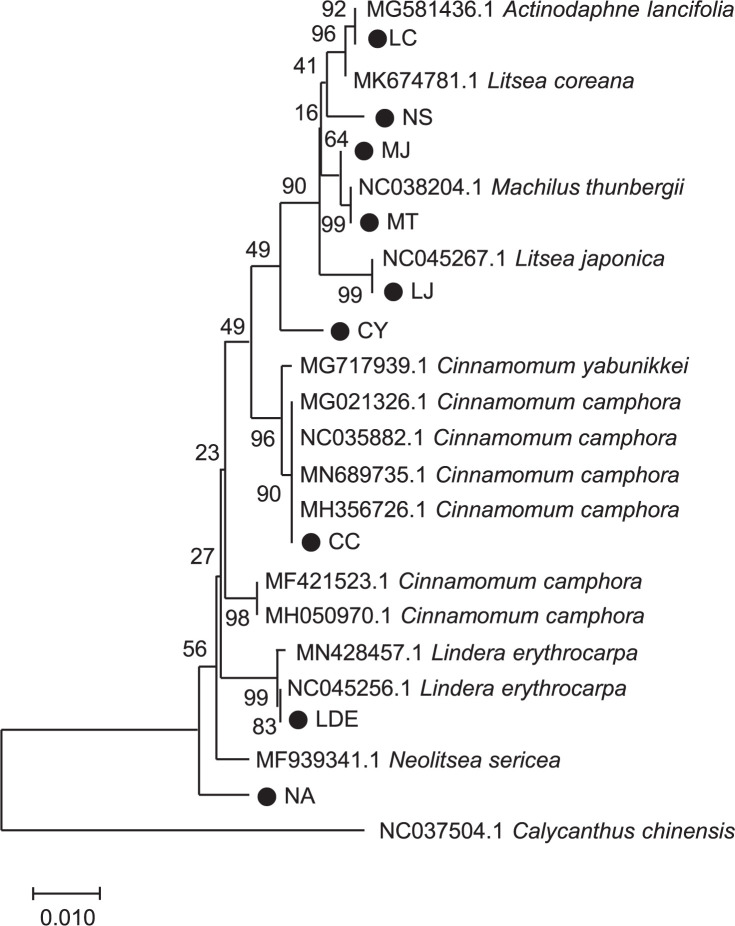
Phylogenetic relationship of Lauraceae samples. Combined sequences of two universal barcoding regions, *trnH-GUG* and *rbcL*, from each sample were used to draw a neighbor-joining tree with 1000 bootstrap replicates. LDE, *Lindera erythrocarpa* Makino; LJ, *Litsea japonica* (Thunb.) Jussieu; NS, *Neolitsea sericea* (Blume) Koidz.; MT, *Machilus thunbergii* Siebold & Zucc.; CC, *Cinnamomum camphora* (L.) J. Presl; CY, *C*. *yabunikkei* H. Ohba; NA, *N*. *aciculata* (Blume) Koidz.; MJ, *M*. *japonica* Siebold & Zucc.; and LC, *L*. *coreana* H.Lév.

Genomic information of each species was obtained from two universal barcoding regions in the plastid genome, *trnH-GUG* and *rbcL*. In the phylogenetic tree, most of our sequences were properly clustered with publicly available plastid genome sequences (Fig 8). Intraspecific diversity due to habitat isolation could have affected the escape of our CY sequence from the CY and CC groups because the published CY sequence was collected in southern China and our CY sequence was collected in Korea. The genetic phylogenetic relationship showed a different topology from that of the chemical-based phylogenetic relationship. A phylogenetic tree based on plastid genome information is widely used for constructing evolutionary relationships between species [48, 49] because of the good converseness of this genetic material across the plant lineage and a high resolution for interspecific diversity. However, secondary metabolites in plants are produced from complex biological pathways that involve multiple genes and are influenced by both genetic and environmental factors. For example, at least twelve catalyzing steps are needed for the biosynthesis of the ginsenoside backbone, and more enzymes (mainly UDP glycosyltransferase) are required in the branching step to produce approximately 20 kinds of different ginsenosides [50, 51]. Moreover, these genes are usually involved in multiple steps and controlled under precise and systematic regulation [52]. Therefore, metabolite-based relationships can differ even in genetically close species because plant metabolites are not made simply by the translation of genetic information but through communication with the environment via complex and diverse processes.

## Conclusions

In the present study, the primary and secondary metabolites of nine Lauraceae species collected in Korea were compared and classified using chemometric multivariate analysis and molecular networking. Multivariate analyses were performed on LC-MS spectral data of the samples to generate PCA and PLS-DA score plots, between which the nine samples were clustered more clearly on the PLS-DA plot. Fourteen important metabolites were selected based on the VIP scores of the PLS-DA model (VIP score > 2.5). The fourteen selected metabolites were also scattered on the PLS-DA loading plot, indicating that these metabolites contributed to discriminating the nine Lauraceae samples. Eleven of the fourteen metabolites were annotated as cinnamic acid derivatives, flavonoids, and isoquinoline alkaloids by using prep-MS or MS/MS fragment pattern analysis. In addition, the chemical diversity of the nine samples was analyzed through molecular network analysis, whereby isoquinoline alkaloids, flavonoids, and lignan clusters were assigned as major clusters. Molecular network analysis facilitated interpretation of the grouping patterns in chemical dendrograms, indicating that the chemical differences between NS and the other samples derive from a high isoquinoline alkaloid content, whereas those between the CC and MJ groups derive from a high lignan content. This approach enabled us to compare the complete chemical composition of several Lauraceae samples simultaneously and interpret the clustering pattern of the chemical dendrogram at the metabolite level.

## Supporting information

S1 File (DOCX)

S1 Data (CSV)
