# Supplementary material for: A comparative phytochemical study of nine Lauraceae species by using chemometric data analysis
Source: PLoS One. 2022 Sep 9;17(9):e0273616. doi: 10.1371/journal.pone.0273616 (PMC9462775; doi:10.1371/journal.pone.0273616)
Supplement: S1 File — (DOCX) [file pone.0273616.s001.docx]

**A comparative phytochemical study of nine Lauraceae species by using** **chemometric data analysis**

Mira Oh1¶, Hyun-Seung Park2¶, Soohyun Um^1^, Tae-Jin Yang^2^, and Seung Hyun Kim^1*^

^1^ College of Pharmacy, Yonsei Institute of Pharmaceutical Sciences, Yonsei University, Incheon, Korea

^2^ Department of Agriculture, Forestry and Bioresources, Research Institute of Agriculture and Life Sciences, and Plant Genomics and Breeding Institute, College of Agriculture and Life Sciences, Seoul National University, Seoul, Republic of Korea

^*^Corresponding author

Seung Hyun Kim

Tel : +82-32-749-4514

Fax : +82-32-749-4105

E-mail : [kimsh11@yonsei.ac.kr](mailto:kimsh11@yonsei.ac.kr)

¶These authors contributed equally to this work.


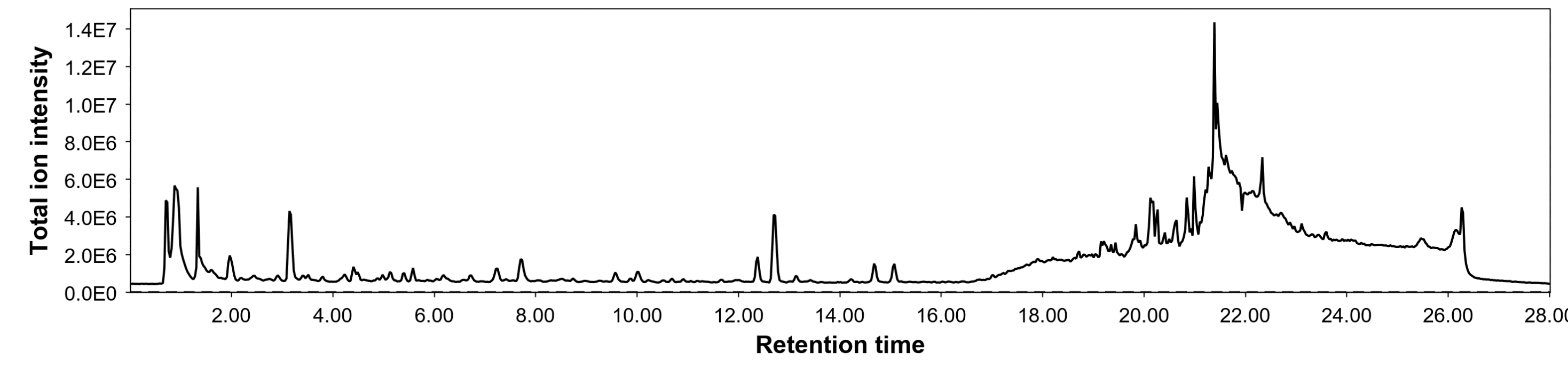


LDE


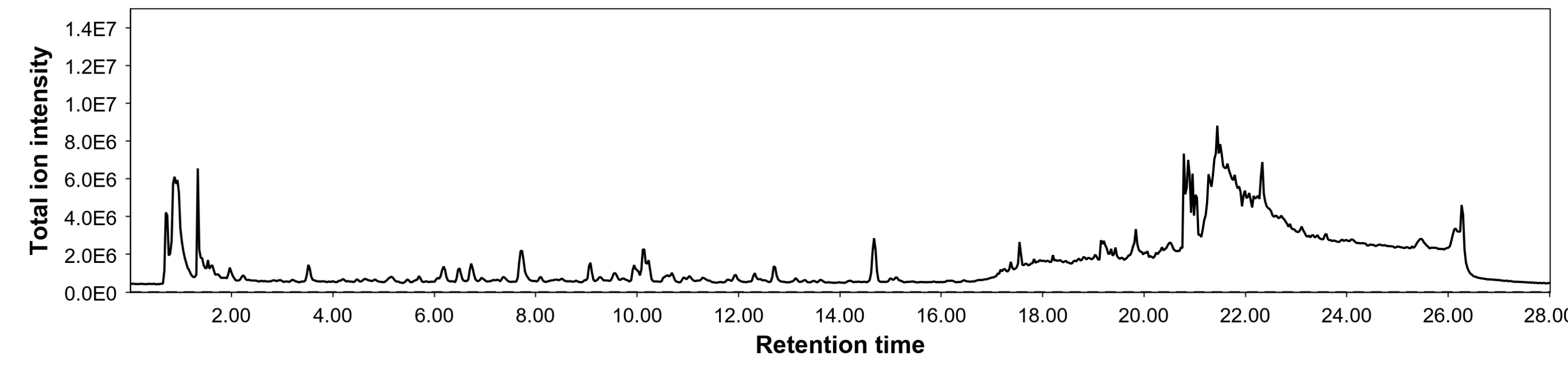


LJ


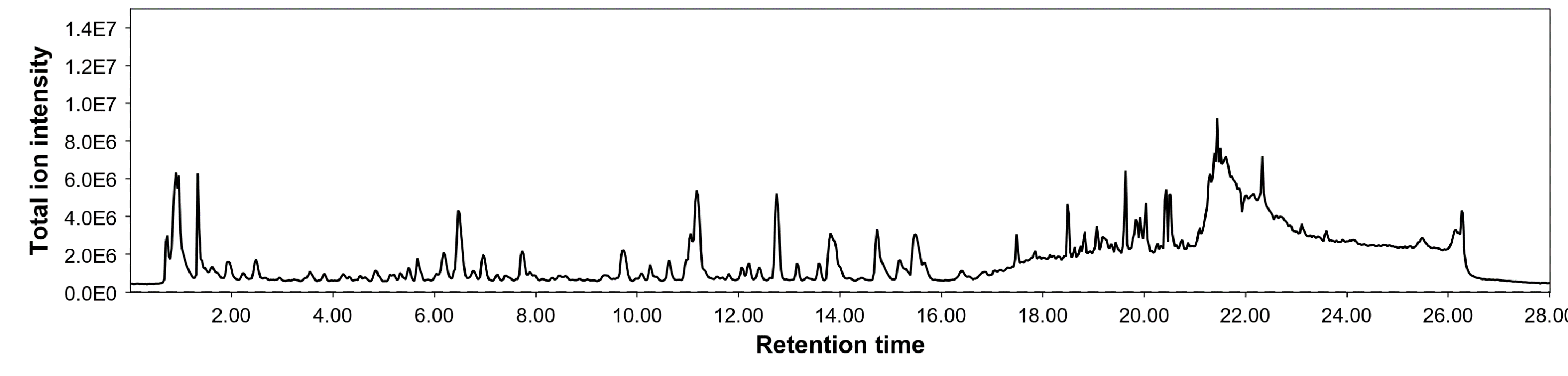


NS


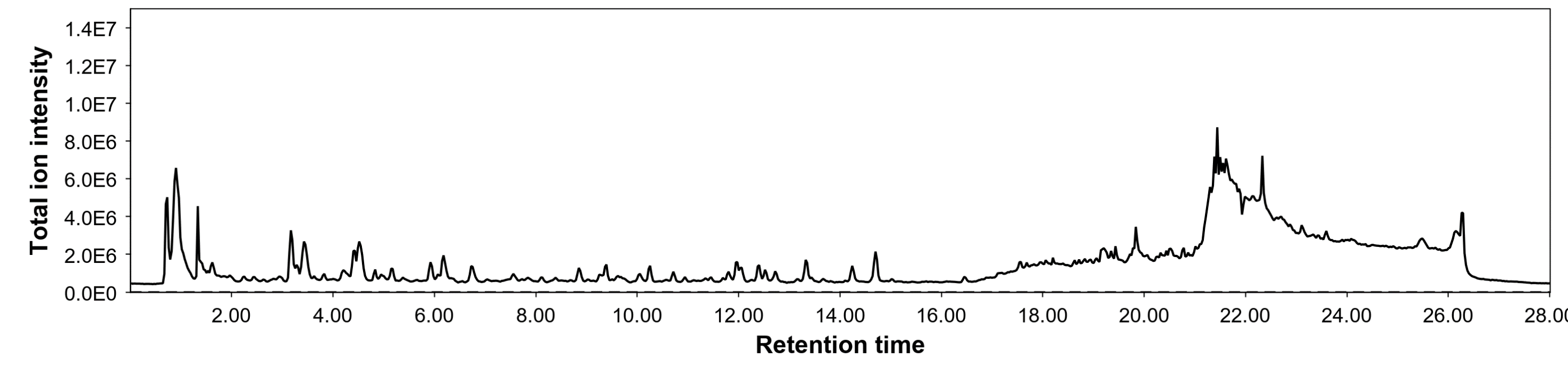


MT


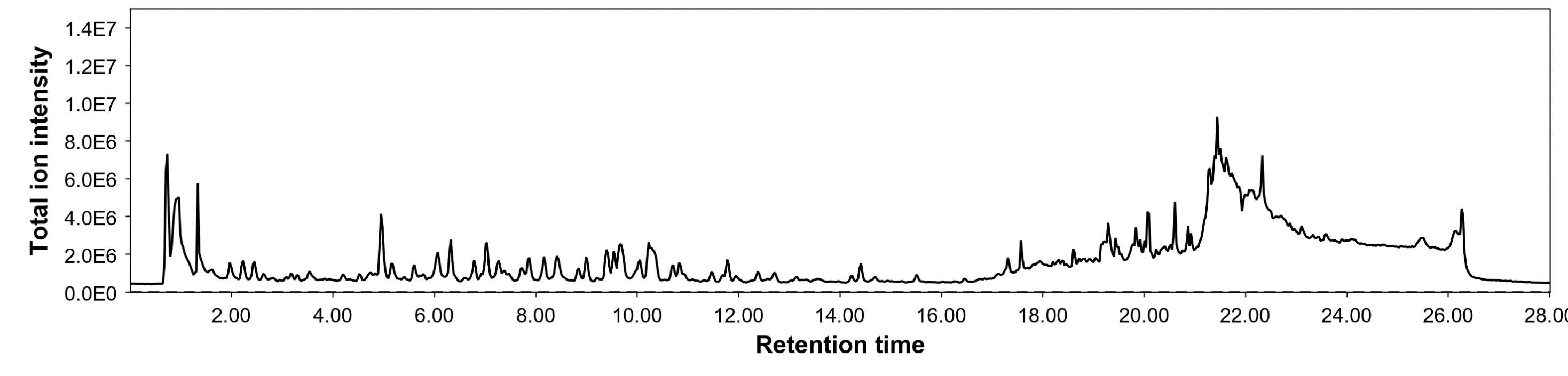


CC


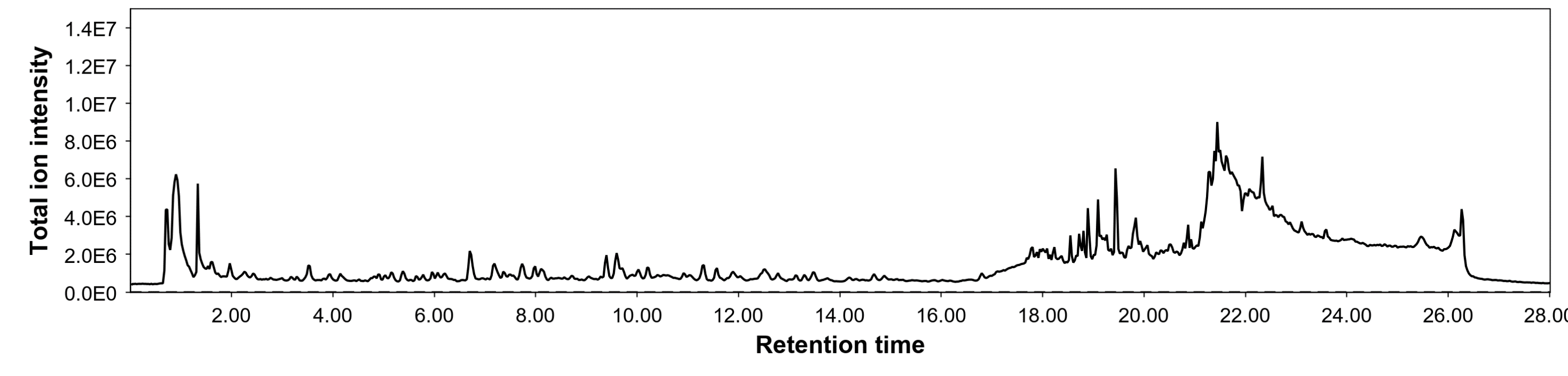


CY


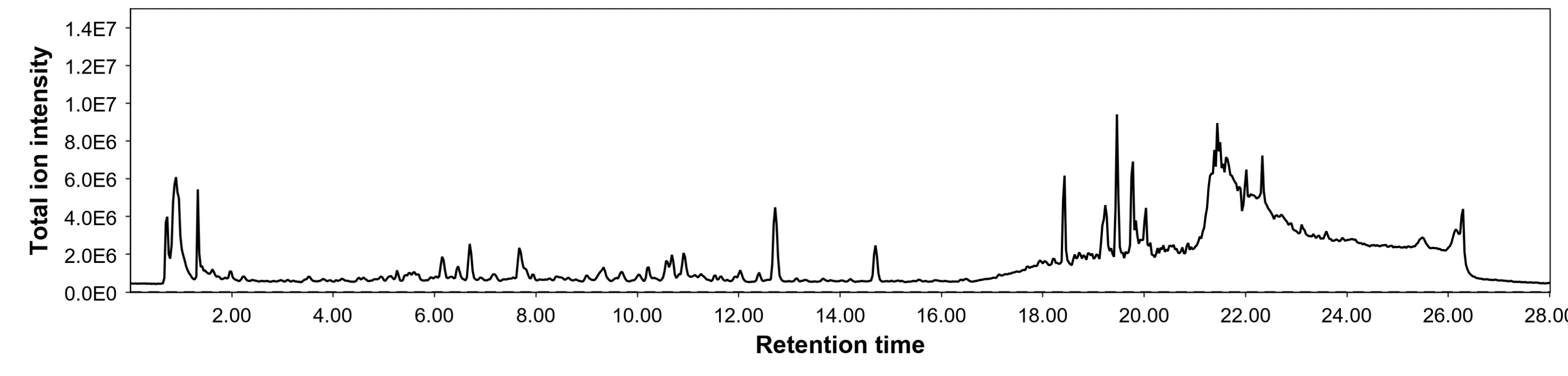


NA


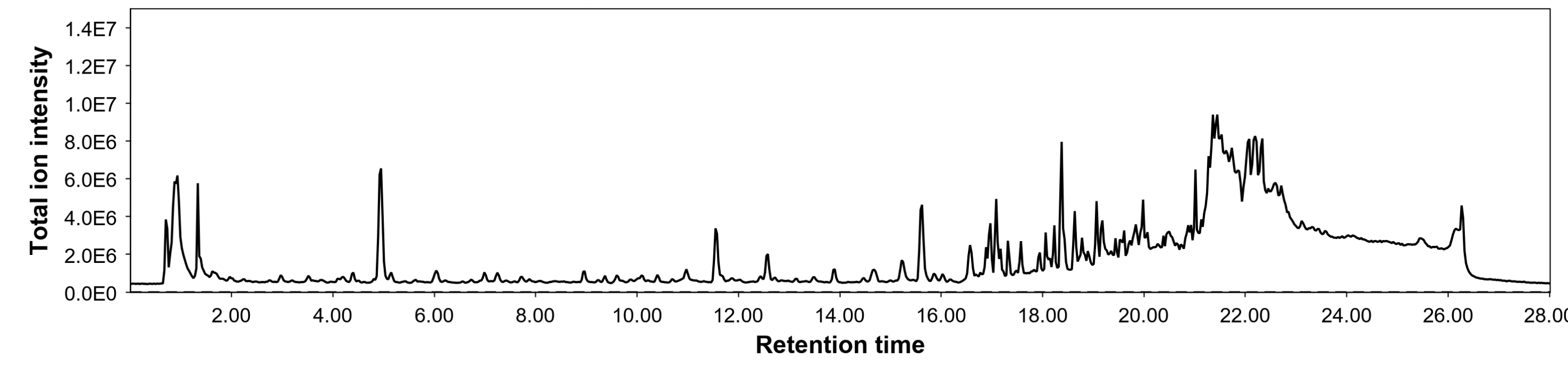


MJ


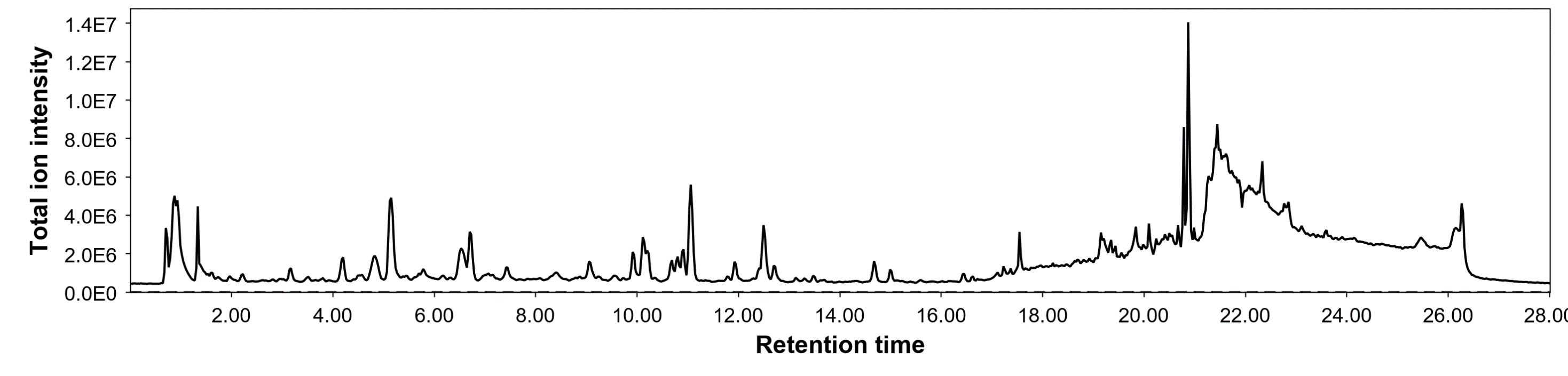


LC

**I**

**H**

**G**

**F**

**E**

**D**

**C**

**B**

**A**

**S1 Fig.** **Representative LC-MS chromatographic profiles of nine Lauraceae species in the positive modes.** (**A**) *Cinnamomum camphora* (L.) J.Presl (CC); (**B**) *C. yabunikkei* H.Ohba (CY); (**C**) *Lindera erythrocarpa* Makino (LDE); (**D**) *Litsea coreana* H.Lév. (LC) ; (**E**) *L. japonica* (Thunb.) Jussieu (LJ); (**F**) *Machilus japonica* Siebold & Zucc. (MJ); (**G**) *M. thunbergii* Siebold & Zucc; (MT). (**H**) *Neolitsea aciculata* (Blume) Koidz. (NA); and (**I**) *N. sericea* (Blume) Koidz. (NS).

**
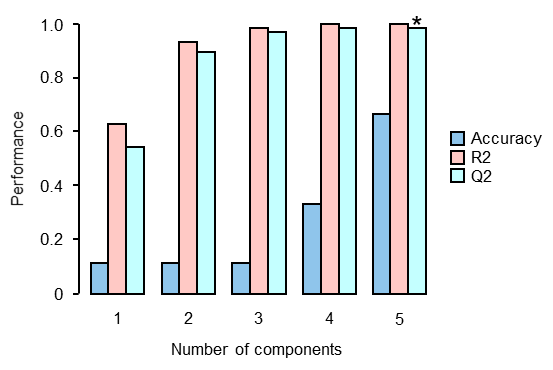
**

**S2 Fig. PLS-DA classification using different number of components.** The star symbol indicates the best classifier (Q2=0.985).


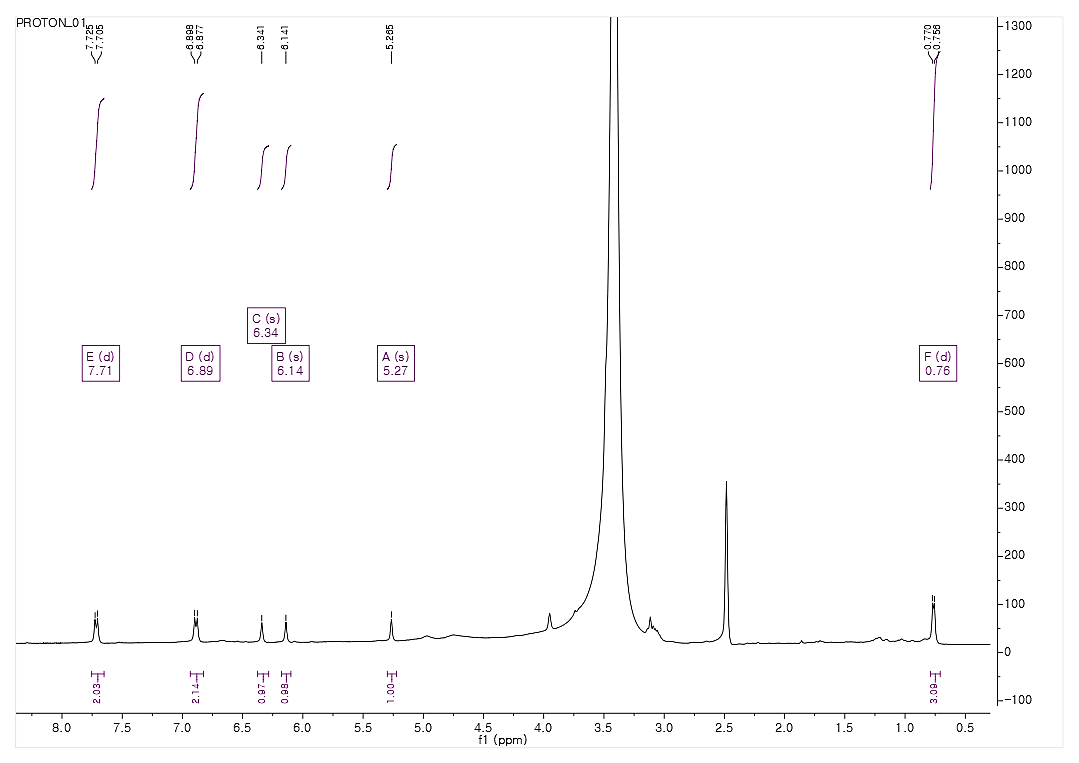


**S3 Fig. ^1^H-NMR spectrum of compound 2 (600 MHz, DMSO-*d_6_*).**


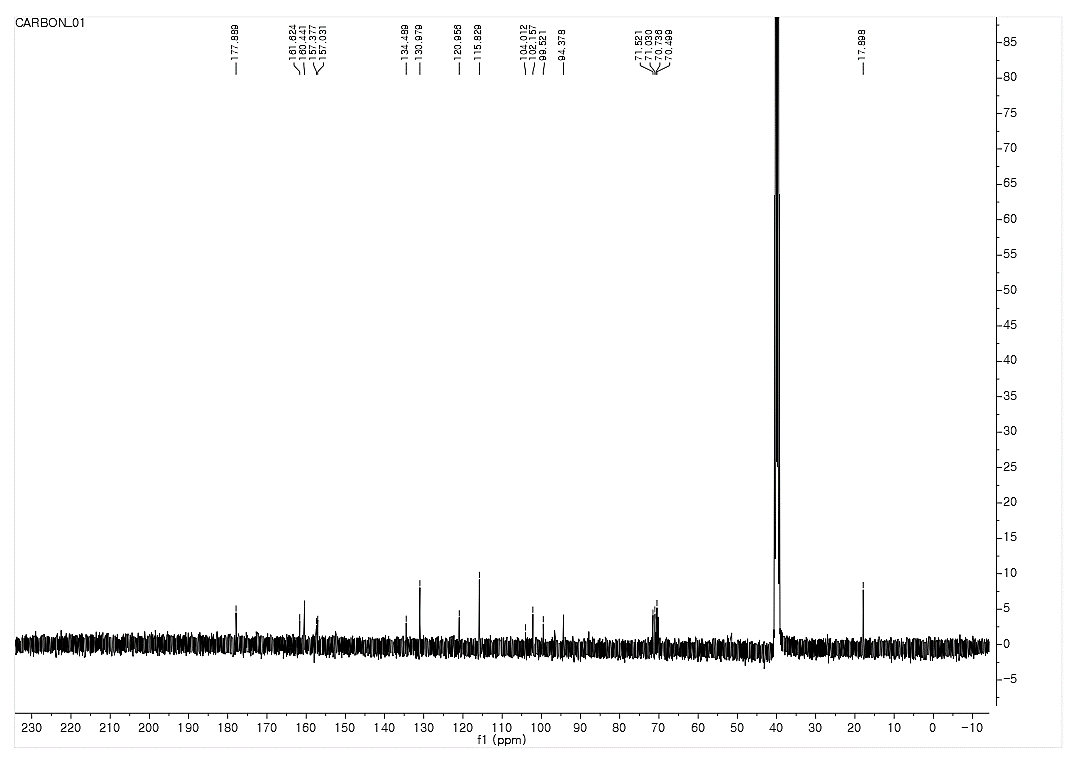


**S4 Fig. ^13^C-NMR spectrum of compound 2 (150 MHz, DMSO-*d_6_*).**


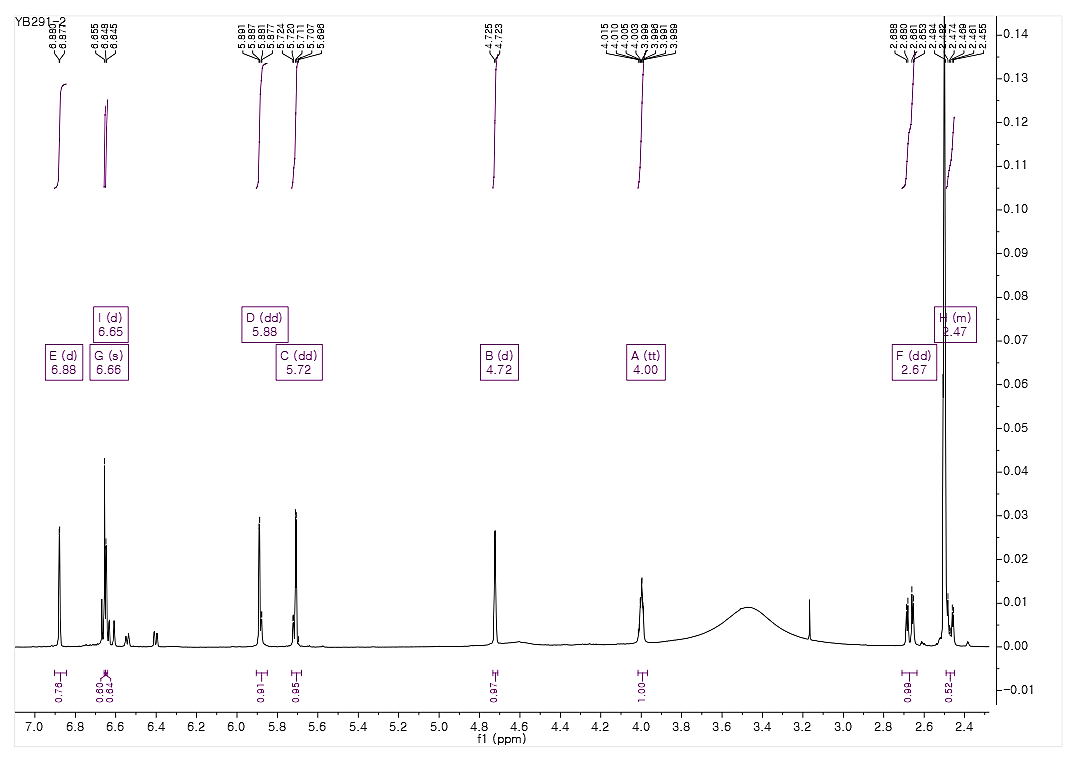


**S5 Fig. ^1^H-NMR spectrum of compound 4 (600 MHz, DMSO-*d_6_*).**


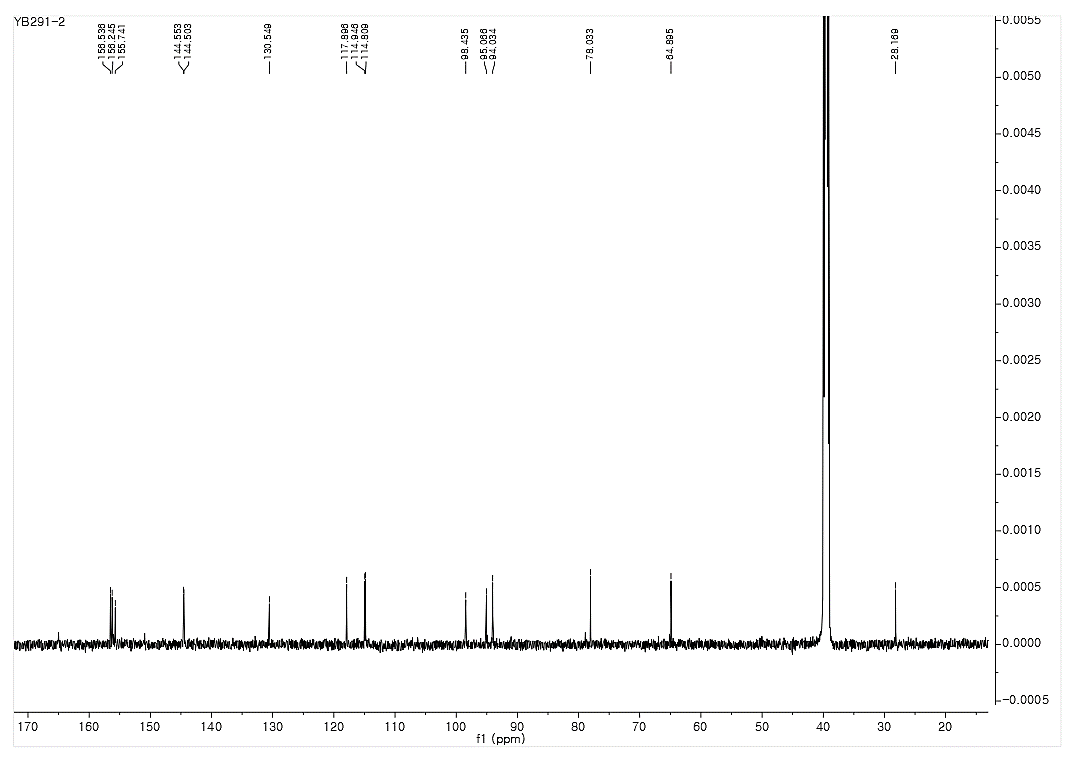


**S6 Fig. ^13^C-NMR spectrum of compound 4 (150 MHz, DMSO-*d_6_*).**


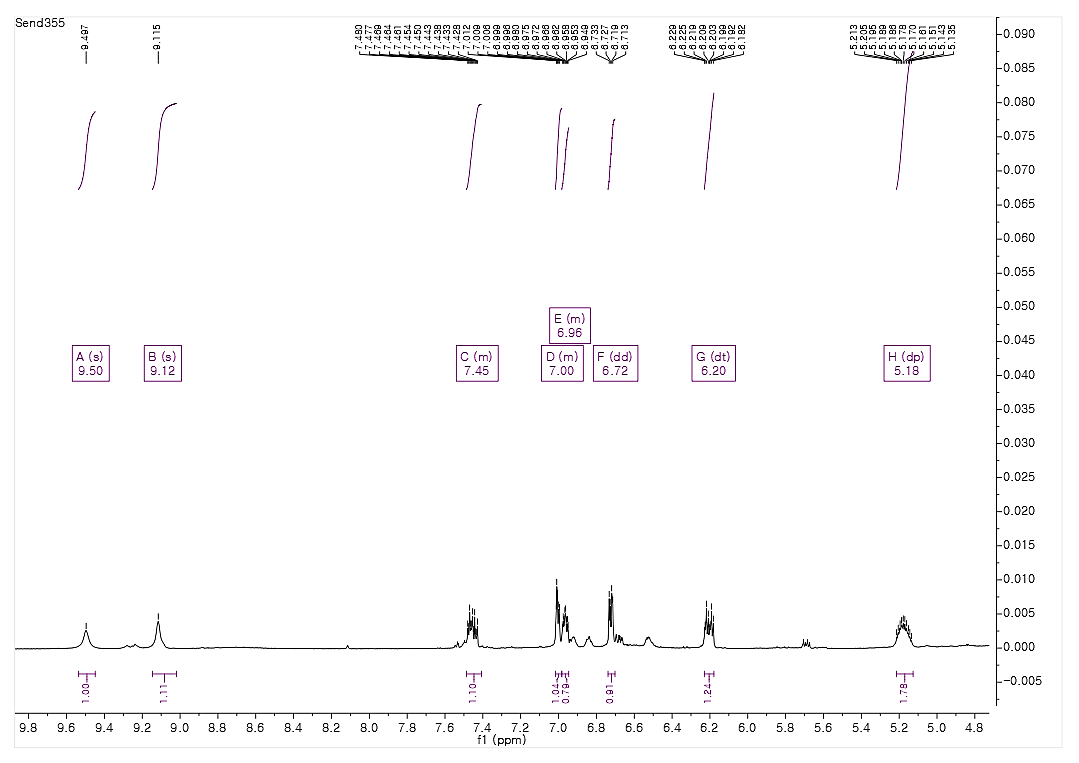


**S7 Fig. ^1^H-NMR spectrum of compound 6 (600 MHz, DMSO-*d_6_*).**


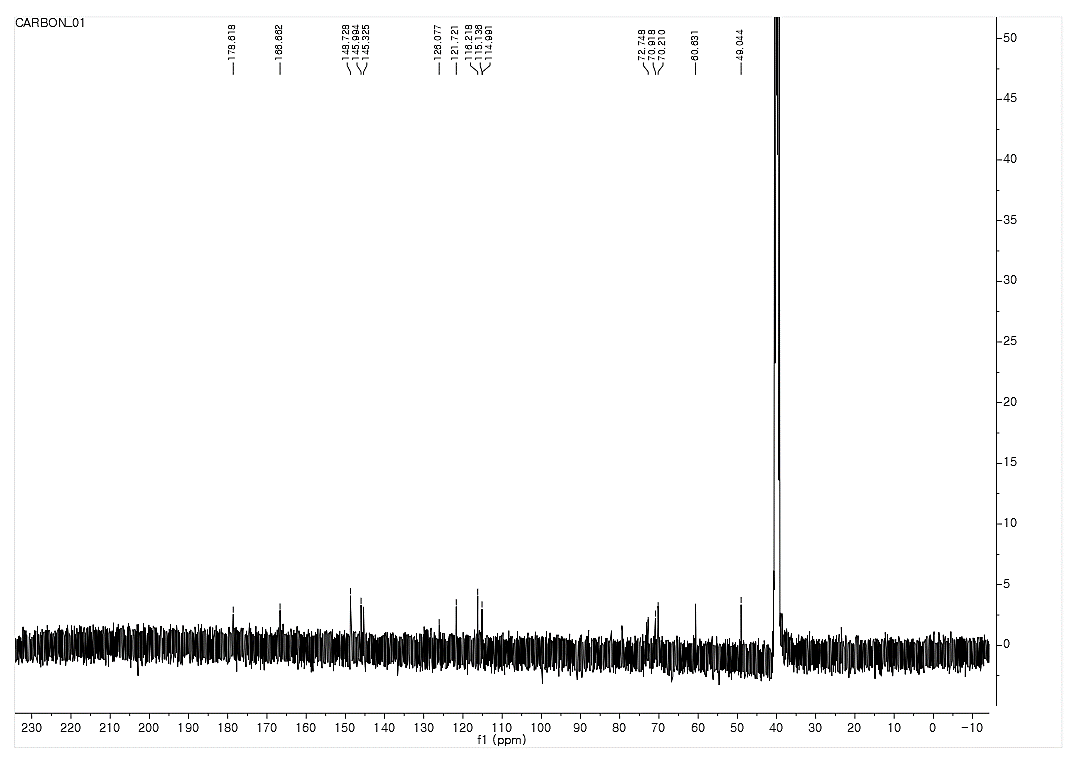


**S8 Fig. ^13^C-NMR spectrum of compound 6 (150 MHz, DMSO-*d_6_*).**


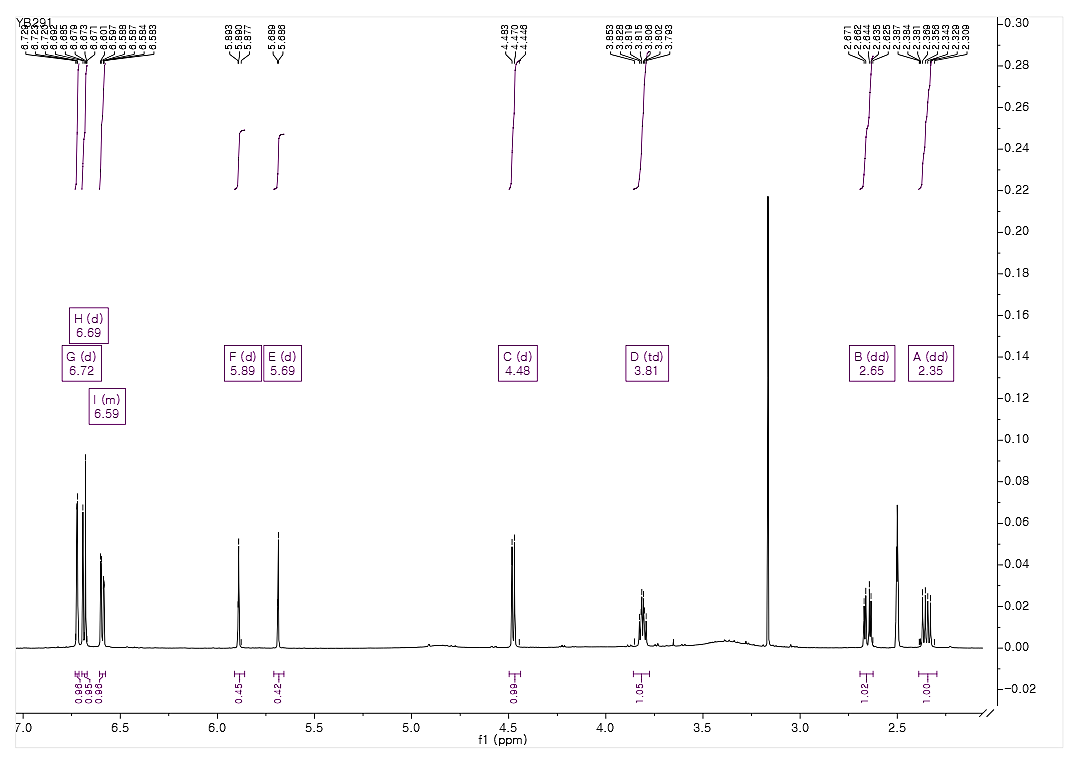


**S9 Fig. ^1^H-NMR spectrum of compound 9 (600 MHz, DMSO-*d_6_*).**


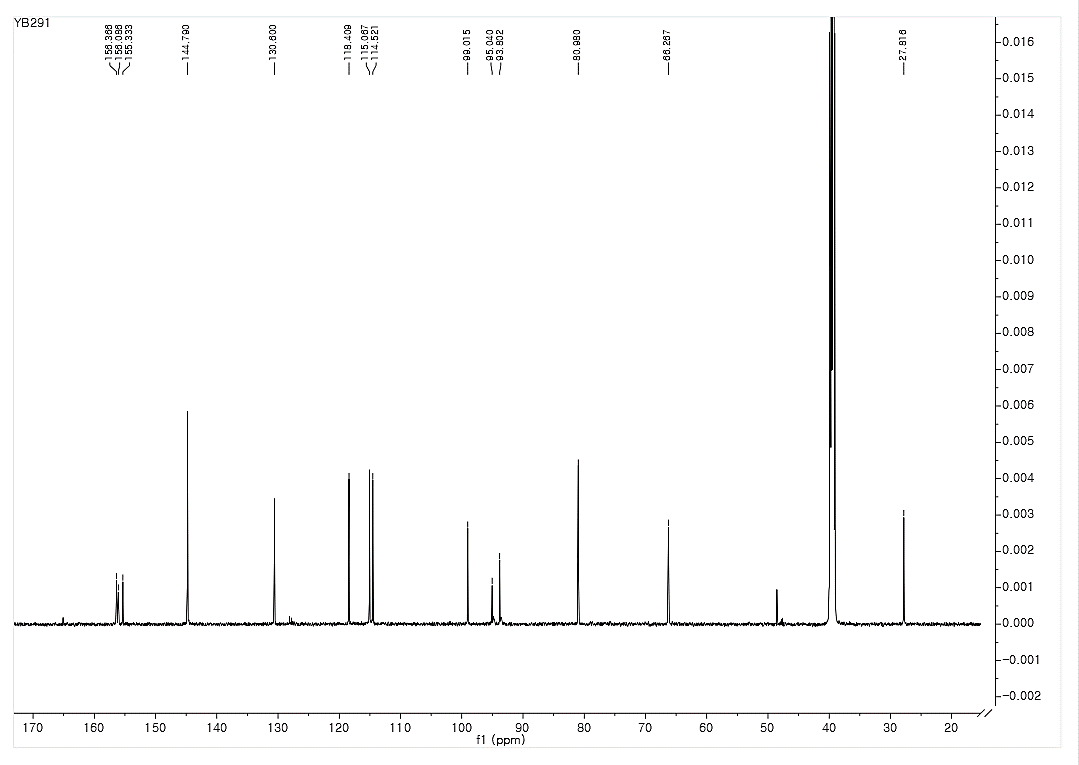


**S10 Fig. ^13^C-NMR spectrum of compound 9 (150 MHz, DMSO-*d_6_*).**


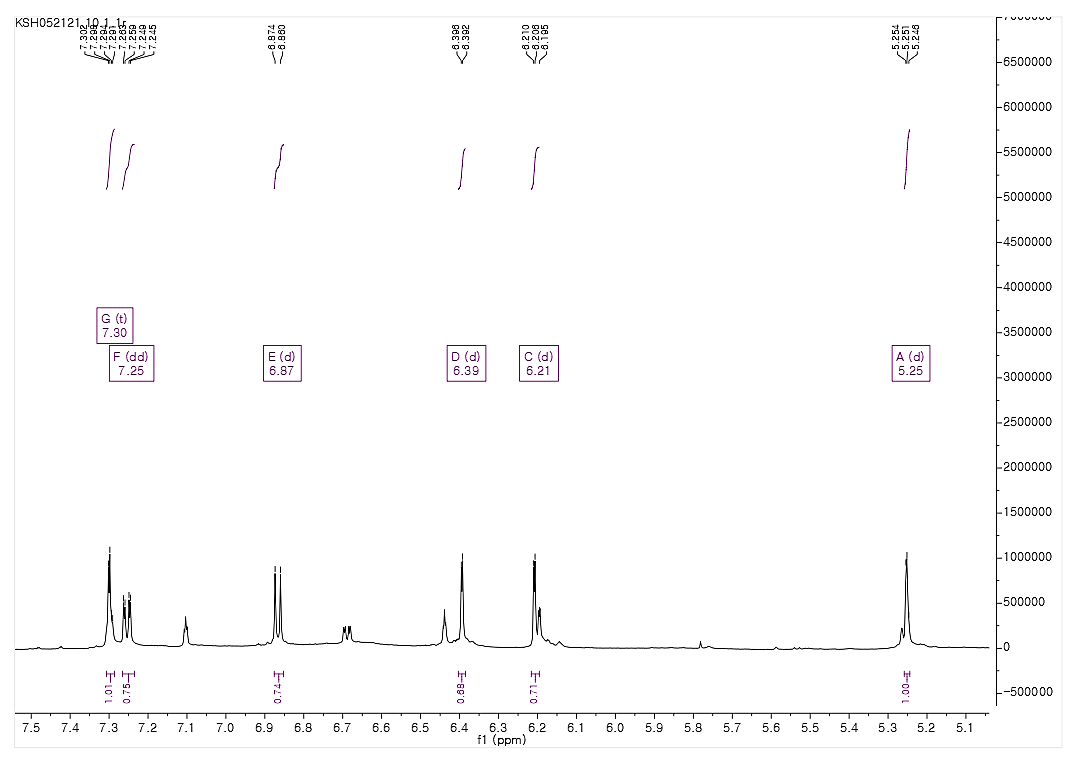


**S11 Fig. ^1^H-NMR spectrum of compound 13 (600 MHz, DMSO-*d_6_*).**


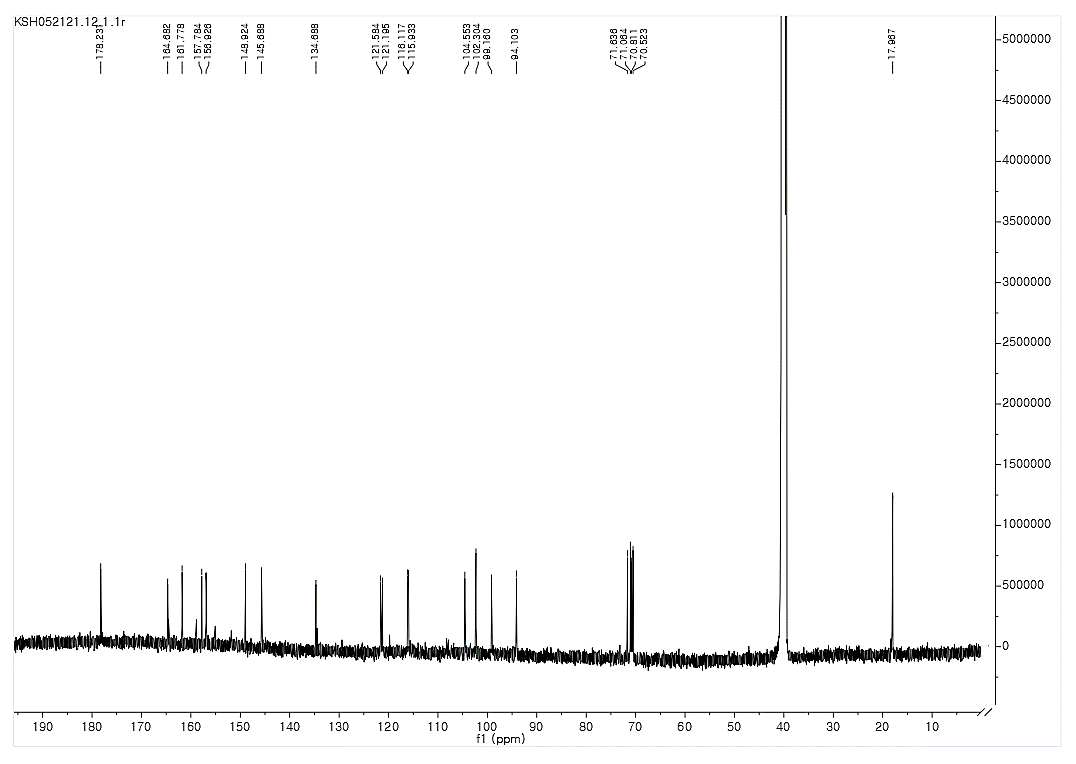


**S12 Fig. ^13^C-NMR spectrum of compound 13 (150 MHz, DMSO-*d_6_*).**

compound **1**

**B**

**A**

compound **6**

**S13 Fig. The LC-MS extracted-ion chromatogram (EIC).** (**A**) EIC of compound **1** (*m*/*z* 355.0999, 3.42 min), which was generated from TIC of *Lindera erythrocarpa* Makino. (**B**) EIC of compound **6** (*m*/*z* 355.1026, 5.40 min), which was generated from TIC of *Machilus japonica Siebold & Zucc.* EIC was obtained using MassHunter qualitative analysis software B.06.00. Both samples were analyzed under identical LC-MS conditions.
